# Dr. Terri Grodzicker: leading *Genes & Development* through 35 years of rapidly advancing molecular biology research

**DOI:** 10.1101/gad.350466.123

**Published:** 2023-01-01

**Authors:** Stephen T. Smale

**Affiliations:** Howard Hughes Medical Institute, Department of Microbiology, Immunology, and Molecular Genetics, Molecular Biology Institute, University of California, Los Angeles, Los Angeles, California 90095, USA

I am honored to have the opportunity to express my deepest appreciation for Dr. Terri Grodzicker's monumental role in leading the scientific journal *Genes & Development* through its formative years and, subsequently, through dramatic ongoing changes in molecular biology research and in the scientific publication landscape. Notably, Terri's contributions to science have extended well beyond *G&D* as a result of her own research and her diverse roles at Cold Spring Harbor Laboratory that have further served the biological research community. Most significant for those external to CSHL is Terri's participation in the oversight of an impressive range of CSHL meetings and laboratory courses. For several decades, CSHL meetings and courses have had a major national and international impact through the dissemination of knowledge and experimental skills.

As a tribute to Terri's contributions, I offer a brief historical perspective that draws on my own experiences interacting with Terri in various capacities, including my role as a member of the *G&D* Editorial Board for more than 20 years and as an author of papers published in *G&D*. Like most historical accounts, my perspective extends beyond facts to personal experiences, but my experiences are undoubtedly consistent with those of many who have had the pleasure of interacting with Terri over the years.

The founding of *G&D* in 1987, followed soon thereafter by the appointment of Terri as the journal's Editor, coincided with the early stages of an explosion in the use of modern molecular biology techniques—including DNA cloning and a number of practical methods for the manipulation of nucleic acids and proteins—to elucidate the mechanisms controlling fundamental molecular processes in viruses and in prokaryotic and eukaryotic cells and organisms. Many of the key molecular biology techniques that drove this explosion were developed in the 1970s, but the expertise required to put them to use was initially limited to a small number of labs, with a dramatic expansion through the 1980s and continuing into the 1990s. The more recent genomics revolution and nascent CRISPR revolution further expanded the capacity to uncover mechanistic principles.

Prior to the launch of *G&D*, many of the most significant advances in molecular biology research were published in two general scientific journals that had published important research in diverse disciplines for more than a century, *Nature* and *Science*, along with a third journal, *Cell*, founded in 1974, just before modern molecular biology research began its largest expansion. Molecular biology advances were also published in other highly respected journals, most notably two journals launched in the early 20th century, the *Journal of Biological Chemistry* and the *Proceedings of the National Academy of Sciences*, along with *Molecular and Cellular Biology*, established by the American Society for Microbiology in 1981. These latter journals were and remain highly respected, but they published relatively large numbers of papers each month (e.g., at the time, a typical issue of the smallest journal, *MCB*, included roughly 50 articles). In this publication landscape, the launch of *G&D* provided an opportunity to highlight a limited number of highly meritorious advances in the growing molecular biology field.

Terri's leadership was instrumental in establishing and solidifying *G&D*’s standards and reputation. The journal focused on fundamental mechanistic advances relevant to a broad range of molecular processes, including the mechanisms and regulation of gene transcription, pre-mRNA splicing, DNA replication, mRNA stability, polyadenylation, recombination, and transposition, with chromatin structure and nuclear organization covered to an increasing extent beginning in the second half of the 1990s. Published studies covered a broad range of physiological processes and took advantage of diverse genetic model organisms and other experimental systems amenable to molecular and biochemical analyses. When genome sequences became available early in the 21st century, genomic studies were welcomed by the journal when used to address important mechanistic questions.

I was honored to serve on the *G&D* Editorial Board beginning in 2001, and in this capacity I developed a strong appreciation for Terri's talents as an Editor. Terri's talents are due in part to her impressive intellect and unique background for an Editor of a major journal. Most leading journals have traditionally been guided either by professional Editors or by active researchers serving as Editors for a limited time as a professional service activity. Terri is rare; as a dedicated leader of a major journal who had successfully run her own lab for many years. Terri's style includes a strong respect for the insights of expert advisors and reviewers, but with an equally strong appreciation for manuscript authors and for the value to the scientific community of publishing meritorious research as rapidly as possible. Terri relies heavily on the opinions of expert reviewers who evaluate the novelty, impact, and rigor of a study. As an author, I did not always agree with the reviewers’ critiques of my lab's papers or with Terri's decision not to publish a subset of them. However, I found *G&D* reviews to be consistently fair and insightful, and I had confidence that the reviews reflected the honest opinions of experts in my fields of study.

Notably, when a study is deemed to be compelling and suitable for the *G&D* audience, Terri has understood that the long lists of additional experiments sometimes recommended by individual reviewers could be detrimental to the advancement of science due to the long, accompanying delays in disseminating important advances to the research community. Terri has been able to effectively make the key distinctions between requested experiments needed to establish the validity of a study's key conclusions and requests that reflect a reviewer's overreach or natural curiosity to know more. Striking an appropriate balance between disseminating research findings as quickly as possible to advance a field and requiring additional experiments prior to publication is a critical skill required of all journal Editors. From my experiences as both a reviewer and author, Terri's skills in finding a suitable balance are rare.

I will briefly expand on my perspective of the importance of *G&D* to two fields that have been the focus of my own lab: eukaryotic transcriptional regulation and molecular immunology. Prior to the launch of *G&D*, the eukaryotic transcriptional regulation field had witnessed a large number of advances, including but not limited to (1) the identification of the three RNA polymerases present in most eukaryotes, (2) the development of cell-free transcription assays that led to the discovery of the core general transcription factors, and (3) the discovery of transcriptional activators and repressors that bind to specific DNA sequences in the promoters and enhancers of eukaryotic genes and regulate transcription initiation in a gene-specific manner. Although difficult to appreciate in 2023, only a small handful of sequence-specific DNA-binding proteins had been reported when *G&D* was launched, and genes encoding DNA-binding proteins had been isolated for only a few.

Thus, there was much to learn through the end of the 1980s and in subsequent decades. Importantly, as more and more transcription factors were being discovered and their genes cloned, Terri wisely chose not to focus *G&D* on reporting the discovery of each new transcription factor unless the study provided a broad mechanistic advance toward elucidating fundamental mechanisms of transcriptional control or revealed an important mechanism by which a specific transcription factor regulates a developmental/physiological process. With respect to fundamental mechanisms of transcription initiation and regulation, early studies focused heavily on the properties and diverse structural organization of promoters and enhancers, the mechanisms by which enhancers communicate with promoters, the diverse mechanisms by which transcriptional regulators communicate with each other and with the general transcription machinery, and the mechanisms by which transcription factor activities are regulated to coordinate developmental and physiological processes. Coactivators and corepressors soon became a major focus of mechanistic research, along with the mechanisms by which nucleosome remodeling and the post-translational modification of histones and other chromatin proteins regulate gene transcription. At a personal level, I paid (and continue to pay) close attention to articles published in *G&D* because the journal has published an unusually large number of major mechanistic advances closely aligned with my own interests in transcriptional regulatory mechanisms.

*G&D* has been similarly important for the broad dissemination of advances in the molecular immunology field. A prominent focus of the field has been on the mechanisms by which signaling pathways, transcription factors, and transcriptional control regions regulate both immune cell development and the rapid activation of immune cells during an immune response. Immune cells have features that, beginning in the 1980s, made them highly attractive for fundamental studies of gene regulation during both cell development and cell activation. These features included the availability of cell lines maintained at defined stages of cell development (most notably for B-cell development), the relative ease with which primary cell populations at different stages of development and activation could be isolated and sorted from the blood and tissues of mice and other animals, and the ability to obtain large numbers of primary immune cells in relatively pure form for molecular interrogation. Because of these features, mechanistic studies of signaling and gene regulation during immune cell development and activation often have relevance that extends beyond the immune system. This has made *G&D* an attractive journal in which to publish mechanistic molecular immunology studies toward the goal of reaching a broader molecular biology audience.

Finally, *G&D* became an important repository for articles focused on genetic events that are unique to cells of the adaptive immune system; namely, V(D)J recombination, immunoglobulin class switch recombination, and somatic hypermutation. These genetic events are of broad interest to the molecular biology community, as they represent fascinating mechanisms that evolved in vertebrates specifically for the purpose of responding to infectious agents. Publication of some of the strongest mechanistic research in these important areas in *G&D* has made it accessible far beyond the immunology field.

To conclude, the importance of Dr. Terri Grodzicker's skillful leadership of *G&D* through 35 years of the molecular biology field's exceptionally rapid advancement cannot be overstated. The molecular biology community owes a debt of gratitude to Terri for her hard work and her insightful and colossal contributions.

